# 15-PGJ_2_, but not thiazolidinediones, inhibits cell growth, induces apoptosis, and causes downregulation of Stat3 in human oral SCCa cells

**DOI:** 10.1038/sj.bjc.6600618

**Published:** 2002-12-02

**Authors:** N G Nikitakis, H Siavash, C Hebert, M A Reynolds, A W Hamburger, J J Sauk

**Affiliations:** Department of Diagnostic Sciences and Pathology, University of Maryland, Baltimore, Maryland, MD 21201, USA; Department of Periodontics, University of Maryland, Baltimore, Maryland, MD 21201, USA; Department of Pathology, University of Maryland, Baltimore, Maryland, MD 21201, USA; Greenebaum Cancer Center, University of Maryland, Baltimore, Maryland, MD 21201, USA

**Keywords:** PPARγ, Stat3, prostaglandin J_2_, thiazolidinediones, oral squamous carcinoma

## Abstract

Activation of peroxisome proliferator-activated receptor gamma (PPARγ) has been linked to induction of differentiation, cell growth inhibition and apoptosis in several types of human cancer. However, the possible effects of PPARγ agonists on human oral squamous cell carcinoma have not yet been reported. In this study, treatment with 15-deoxy-Δ^12,14^-PGJ_2_ (15-PGJ_2_), a natural PPARγ ligand, induced a significant reduction of oral squamous cell carcinoma cell growth, which was mainly attributed to upregulation of apoptosis. Interestingly, rosiglitazone and ciglitazone, two members of the thiazolidinedione family of PPARγ activators, did not exert a growth inhibitory effect. Given the critical role that the oncogene signal transducer and activator of transcription 3 (Stat3) plays in head and neck carcinogenesis, its potential regulation by PPARγ ligands was also examined. Treatment of oral squamous cell carcinoma cells with 15-PGJ_2_ induced an initial reduction and eventual elimination of both phosphorylated and unphosphorylated Stat3 protein levels. In contrast, other PPARγ did not induce similar effects. Our results provide the first evidence of significant antineoplastic effects of 15-PGJ_2_ on human oral squamous cell carcinoma cells, which may be related to downmodulation of Stat3 and are at least partly mediated through PPARγ-independent events.

*British Journal of Cancer* (2002) **87**, 1396–1403. doi:10.1038/sj.bjc.6600618 www.bjcancer.com

© 2002 Cancer Research UK

## 

Peroxisome proliferator-activated receptors (PPARs) are members of the nuclear hormone receptor family, which function as ligand-dependent, sequence-specific activators of transcription. The PPAR family consists of three distinct molecules, termed α, δ (β, FFAR or NUC-1), and γ, encoded by separate genes and characterised by specific tissue and developmental distribution patterns (Mangelsdorf *et al*, 1995; Lemberger *et al*, 1996; Gelman *et al*, 1999; Kersten *et al*, 2000). A variety of natural and pharmacological ligands, including prostaglandins, the fibrate class of hypolipidaemic drugs, the anti-diabetic drugs thiazolidinediones, and certain non-steroidal anti-inflammatory drugs, are able to bind to and activate PPARs (Forman *et al*, 1995, 1997; Yu *et al*, 1995; Lehmann *et al*, 1997). Upon activation, PPARs heterodimerise with the retinoic X receptor (RXR) and bind to peroxisome proliferator response elements (PPREs), located in the promoter region of target genes, driving their transcription (Mangelsdorf *et al*, 1995).

PPARs were initially described as molecular targets for compounds that induce peroxisome proliferation (Issemann and Green, 1990). However, shortly after their discovery, it became apparent that the physiologic role of PPARs extends far beyond peroxisome proliferation, involving such diverse processes as lipid homeostasis, insulin sensitisation, inflammation, and cell proliferation (Lemberger *et al*, 1996; Gelman *et al*, 1999; Kersten *et al*, 2000). Today, PPARs are recognised as key regulators of lipid homeostasis, playing fundamental roles in adipogenesis and fat catabolism (Lemberger *et al*, 1996; Kersten *et al*, 2000). Potential roles of PPARs in the treatment of diabetes mellitus (Vamecq and Latruffe, 1999), in inflammation control (Gelman *et al*, 1999), and in the regulation of atherosclerosis and thrombosis (Vamecq and Latruffe, 1999; Duez *et al*, 2001) have also been described.

The role of PPARγ in the acquisition of an adipocyte phenotype, through the control of the expression of genes that promote cell cycle withdrawal, drive differentiation and induce apoptosis (Shao and Lazar, 1997; Debril *et al*, 2001), prompted many investigators to study the potential function of PPARγ in neoplasia. Multiple lines of evidence suggest that PPARs, especially PPARγ, play an important role in modulating cell proliferation and tumour growth (Gelman *et al*, 1999; Kersten *et al*, 2000; Debril *et al*, 2001; Park *et al*, 2001; Rosen and Spiegelman, 2001). Ligand-induced PPARγ activation has been shown to promote differentiation and to induce cell growth inhibition and apoptosis in several types of human cancer, including colon cancer (Sarraf *et al*, 1998; Kitamura *et al*, 1999; Yang and Frucht, 2001), breast cancer (Elstner *et al*, 1998; Mueller *et al*, 1998), lung cancer (Chang and Szabo, 2000), prostate cancer (Kubota *et al*, 1998; Butler *et al*, 2000), gastric cancer (Sato *et al*, 2000), liposarcoma (Tontonoz *et al*, 1997; Demetri *et al*, 1999), and leukaemia (Sugimura *et al*, 1999). Histological and biochemical evidence indicate that PPARγ ligands induce tumour cell differentiation in patients with advanced liposarcoma (Demetri *et al*, 1999). Consistent with its possible function as a tumour suppressor gene, PPARγ has been reported to be functionally mutated in sporadic cases of colon cancer (Sarraf *et al*, 1999); nonetheless, the overall incidence of PPARγ mutations in human malignancies seems to be very rare (Ikezoe *et al*, 2001).

We recently showed that the non-steroidal anti-inflammatory drug (NSAID) sulindac induces cell growth inhibition and apoptosis in human oral squamous cell carcinoma (SCCa) cells, accompanied by upregulation of the mRNA and protein expression of PPARγ. Treatment with antisense PPARγ oligonucleotides abolished the cell growth inhibitory effect of the NSAID sulindac. These results suggest that upregulation of PPARγ expression and activation may be, at least partially, responsible for sulindac's antineoplastic effect (Nikitakis *et al*, 2002a). However, it is unknown whether ligand-induced PPARγ activation may alter the cell growth of oral SCCa. Here, we assessed the effects that the natural PPARγ ligand 15-deoxy-Δ^12,14^-PGJ_2_ (15-PGJ_2_) and the synthetic PPARγ ligands rosiglitazone and cigliatazone have on cell growth, apoptosis and cell proliferation in oral SCCa cells. Moreover, we explored the possibility that PPARγ activation may affect the expression and activation of Stat3, an oncogene that plays a critical role in head and neck carcinogenesis (Grandis *et al*, 1998, 2000; Bromberg *et al*, 1999; Bowman *et al*, 2000) and is downregulated by sulindac in oral SCCa cells (Nikitakis *et al*, 2002b).

## MATERIALS AND METHODS

### Cell lines and cell culture

Experiments were performed using established cell lines of human oral SCCa (SCC-4, -9, -15 and -25) obtained from the American Type Culture Collection (ATCC) (Manassas, VA, USA). Cells were cultured in a 1 : 1 mixture of Ham's F12 and Dulbecco's Modified Eagle's Medium (DMEM) containing 10% foetal bovine serum (FBS), 100 units of penicillin, 100 μg ml^−1^ streptomycin and 0.4 μg ml^−1^ hydrocortisone (Sigma Chemical Co., St. Louis, MO, USA) at 37°C in a 5% CO_2_ air atmosphere. Cells were subcultured by disaggregation with trypsin (0.1%)–EDTA (0.01%) in phosphate buffered saline (PBS) at pH 7.5.

### Quantitative reverse transcriptase polymerase chain reaction

To estimate the mRNA levels of *PPAR*γ, real-time quantitative reverse transcriptase polymerase chain reaction (RT–PCR) was performed using a PE Applied Biosystems protocols. Total RNA was isolated using the TRIZOL Reagent (BRL/Life Technologies) and the concentration of RNA was determined using spectrophotometry. The forward and reverse *PPAR*γ primers were selected using Primer Express software (PE Applied Biosysystems, Foster City, CA, USA) as follows: 5′-TATCGACCAGCTGAATCCAGAG-3′ (forward) and 5′-TCGCCTTTGCTTTGGTCA-3′ (reverse). For the PCR reaction, a SYBR® Green PCR kit (PE Applied Biosystems) was used and the analyses were performed in triplicate. For each well, 5 μl of 25 nM RNA extract were added to a solution consisting of: 25 μl Master Mix solution (SYBR Green PCR Buffer, AmpErase® UNG, AmpliTaq Gold® DNA Polymerase, dATP, dCTP, dGTP, dUTP and 25 mM MgCl_2_), 1 μl of RNAse inhibitor, 0.25 μl of reverse transcriptase (MultiScribe), 1 μl of each primer and 16.75 μl of water. The amplification process included 30 min at 48°C, 10 min at 95°C, followed by 40 cycles of 15 s at 95°C and 1 min at 60°C. Thermal cycling and fluorescence detection were performed using an ABI 5700 Prism (PE Applied Biosystems). Relative quantitation of the signal of *PPAR*γ mRNA followed. The signal of the target mRNA was normalised by comparison with the housekeeping gene glyceraldehyde-3-phosphate dehydrogenase (*GAPDH*) mRNA signal. The normalised amount of *PPAR*γ mRNA present in each cell line was calculated by arbitrarily designating SCC25 cells as a calibrator using a comparative Ct method following PE Applied Biosystems protocols.

### Immunocytochemistry

Cells were plated on chamber slides (LabTech Nalge/Nunc) at a density of 5×10^4^ cells/chamber and were allowed to grow until almost confluent. The cells were then rinsed with Hanks' balanced salt solution (HBSS) and fixed with 95% ethanol for 20 min. PPARγ protein expression was ascertained by immunocytochemical analysis (Santa Cruz Biotechnology, Santa Cruz, CA, USA, sc-1984) 1 : 50. The presence of antibody staining was determined after incubation of the sections with a secondary antibody (Biogenex, San Ramon, CA, USA, HK327-UG) 1 : 20, followed by the application of StrepABComplex/HRP (Dako, Carpinteria, CA, USA, K0377), and diaminobenzidine (DAB). The slides were counterstained with Harris's haematoxylin. The intensity of the immunostaining was classified as: 0 (negative), 1 (weakly positive), 2 (moderately positive), and 3 (strongly positive).

### Immunohistochemistry

Ten cases of oral squamous cell carcinoma were randomly selected from the files of the Department of Diagnostic Sciences and Pathology. No patients were identified for these studies. The histological slides were reviewed to confirm diagnosis.

Five-micron sections of paraffin-embedded tissue were mounted on glass slides, deparaffinised and rehydrated. An antigen retrieval procedure was performed by placing the sections in Citra-solution (HK086-9K; Biogenex, San Ramon, CA, USA) inside a plastic pressure cooker, which was positioned in a microwave oven (Kenmore; Sears, Chicago, IL, USA). The specimens were treated by two cycles, 15 min each, at a high level and at level 4, respectively. Endogenous peroxidase activity was blocked with 3% hydrogen peroxide and non-specific protein was blocked with a universal blocking reagent (HK085-5K; Biogenex). Sections were then treated with PPARγ antibody, followed by incubation with secondary antibody, application of StrepABComplex/HRP and staining with diaminobenzidine (DAB) and Harris haematoxylin, using the aforementioned reagents and dilutions.

### Cell growth inhibition

Cells were plated on 24-well plates at a density of 5×10^4^ cells well^−1^. After 24 h, the growth medium was supplemented with DMSO at a concentration of 0.1% or with either one of the following: 15-deoxy-Δ^12,14^-Prostaglandin J_2_ (15-PGJ_2_) (Cayman Chemical, Ann Arbor, MI, USA) at concentrations of 10 or 20 μM, rosiglitazone (Cayman Chemical) at concentrations of 25 or 50 μM, and ciglitazone (Biomol, Plymouth Meeting, PA, USA) at concentrations of 25 or 50 μM. All three compounds were dissolved in 100% DMSO, so that the final concentration of DMSO did not exceed 0.1%. Following incubation for 24, 48 or 72 h, either treated or untreated cells were removed enzymatically and counted using a Coulter Counter (Coulter Model ZI, Coulter Corporation, Miami, FL, USA). The per cent of growth was determined setting as 100% the growth of cells treated only with the vehicle (0.1% DMSO). All analyses were performed in triplicate.

### Cell cycle analysis

Cells were treated either with the vehicle alone (0.1% DMSO) or with 10 or 20 μM of 15-PGJ_2_ dissolved in 100% DMSO for 72 h. Cells were dissociated using trypsin-EDTA in PBS, pelleted and resuspended in 1 ml of PBS. While vortexing, 5 ml of 70% ethanol in distilled water were added dropwise. The cells were incubated at 4°C for 30 min and then were centrifuged at 2500 **g** for 5 min. The pellet was resuspended in 200 μl of two-fold propidium iodide (PI) dye and 200 μl of RNAse A (2 mg ml^−1^) and the tubes were incubated for 45 min in the dark at 4°C. Cells were filtered through nylon mesh prior to analysis. The DNA content of cells stained with PI and was measured with a FACScan instrument using Cell Quest software (Becton/Dickinson). To determine the proportion of cells in G0-G1, S and G2-M, cell cycle analysis of DNA histograms was performed using ModFitLTV2.0 (PMac). All analyses were performed in duplicate.

### Apoptosis analysis

Apoptosis was assessed by Annexin V-FITC staining (BD Biosciences). In essence, cells were treated either with the vehicle alone (0.1% DMSO) or with 10 or 20 μM of 15-PGJ_2_ dissolved in 100% DMSO for 72 h. Then, cells were washed twice with cold PBS and resuspended in 1× binding buffer at a concentration of 1×10^6^ cell ml^−1^. One hundred microlitres of the solution were transferred to a 5 ml culture tube and 5 μl of Annexin V-FITC and 5 μl of PI were added. The mixture was gently vortexed and incubated for 15 min in the dark. Four hundred microlitres of 1× binding buffer were added to each tube and FACScan analysis of the samples was performed within 1 h using a FACS scan equipped with Cell Quest software (Becton Dickenson). The following controls were used to set up compensation and quadrants: (1) unstained cells, (2) cells stained with Annexin V-FITC alone, and (3) cells stained with PI alone. The percentage of cells that have been induced to undergo apoptosis was determined by subtracting the percentage of apoptotic cells in the untreated population from the percentage of apoptotic cells in the treated population. All analyses were performed in duplicate.

### Protein lysate preparation and Western blotting

Cells were plated on 6-well plates at a density of 5×10^4^ cells well^−1^ and were allowed to grow to 80% confluency. Then, 15-PGJ_2_ at 20 μM concentration, rosiglitazone at 50 μM concentration, or ciglitazone at 50 μM concentration, was added to the medium. All three compounds were dissolved in 100% DMSO, so that the final concentration of DMSO did not exceed 0.1%. Following incubation for various time periods, the cells were washed twice with cold PBS, lysed in RIPA buffer (50 mM Tris (pH 7.4), 150 mM NaCl, 1% Triton X-100, 1% deoxycholic acid, sodium salt, 0.1% sodium dodecyl sulphate (SDS), 100 μg ml^−1^ phenylmethylsulphonyl fluoride, 1 μg ml^−1^ aprotinin, 1 mM dithiothreitol, and 1 mM sodium orthovanadate) for 10 min, and scraped. The extracts were centrifuged at 40 000 **g** for 15 min at 4°C. Protein concentrations were measured and equalised using Bio-Rad protein assay (Bio-Rad Laboratories, Richmond, CA, USA) according to the manufacturer's instructions.

Western blot analysis was performed using phospho-Stat3 (Tyr 705) antibody (1:500 dilution) (Cell Signaling Technology, Beverly, MA, USA) according to the manufacturer's instructions. Blots were stripped (20 mM dithiothreitol, 2% SDS, and 67.5 mM Tris-HCl (pH 6.7)) and then reprobed sequentially with Stat3 (Tyr 705) antibody (1 : 1000 dilution) (Cell Signaling Technology, Beverly, MA, USA) and with actin antibody (1 : 500 dilution) (Sigma, Saint Louis, MO, USA).

### Statistical analysis

Data were submitted to an analysis of variance with repeated measures (time and dosage), using Newman–Keuls *post hoc* comparisons where appropriate (Statistica for Windows, StatSoft, Inc., Tulsa, OK, USA). An alpha value of *P*⩽0.05 was used in all models and *post hoc* comparisons.

## RESULTS

### Expression of PPARγ in oral SCCa cell lines and tissues

Quantitative RT–PCR analysis showed mRNA expression for *PPAR*γ in all four oral SCCa cell lines. The lowest levels of *PPAR*γ mRNA were expressed in SCC25 and the highest in SCC9 (Table 1

**Table 1:**
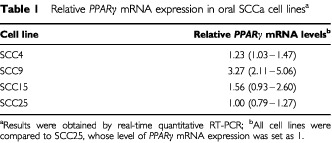
Relative *PPARγ* mRNA expression in oral SCCa cell lines^a^

). Protein expression of PPARγ was detected by means of immunocytochemistry; PPARγ immunostaining, primarily in a cytoplasmic location, was evident in all four oral SCCa cell lines (Figure 1A,B

**Figure 1:**
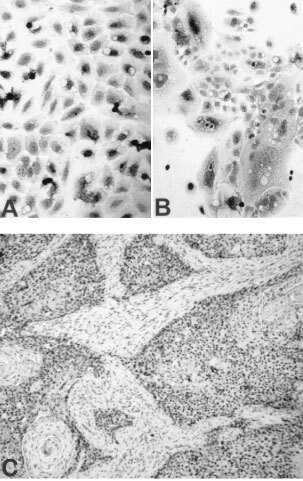
(**A**, **B**) Immunocytochemical detection of PPARγ protein expression in oral SCCa cells (**A**, SCC25; **B**, SCC9). (**C**) Immunohistochemical detection of PPARγ in specimens of oral SCCa; immunostaining was limited to the well-differentiated areas of the tumours.

). Immunohistochemistry for PPARγ in tumour specimens of patients with oral SCCa revealed PPARγ production by the tumour cells, which was limited to the well-differentiated areas of the tumours (Figure 1C).

### Effect of PPARγ agonists on cell growth inhibition

**Figure 2:**
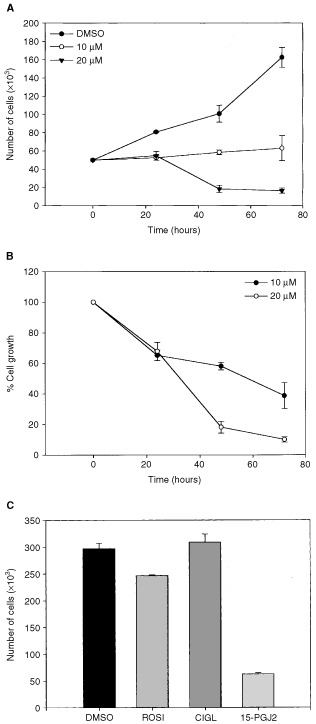
(**A**, **B**) Effect of 15-deoxy-Δ^12,14^-PGJ_2_ on growth of oral SCC25 cells. The cells were treated with 0.1% DMSO, 10 μM 15-deoxy-PGJ_2_ or 20 μM 15-deoxy-PGJ_2_ for various periods of time (24, 48, or 72 h). In (**A**), the real number of cells is depicted, while in (**B**), the per cent of cell growth is shown, after setting the growth of cells treated only with the vehicle as 100%. A statistically significant reduction of cell growth was observed. Similar results were obtained from the other oral SCCa cell lines tested. (**C**) Effect of rosiglitazone (ROSI), ciglitazone (CIGL) and 15-deoxy-Δ^12,14^-PGJ_2_ on growth of oral SCC9 cells. The cells were treated with 0.1% DMSO, 50 μM rosiglitazone, 50 μM ciglitazone or 20 μM of 15-deoxy-PGJ_2_ for 72 h. Only 15-PGJ_2_ induced a significant cell growth inhibition. All tested oral SCCa cell lines gave similar results.

Oral SCC25 cells treated with 10 or 20 μM of 15-PGJ_2_ exhibited reduction in cell growth (Figure 2

); similar results were obtained from the other oral SCCa cell lines. Combining the results from all four cell lines, a significant main effect for time (F_2,6_=20.6, *P*⩽0.01) and interaction of time *vs* dosage (F_4,12_=17.4, *P*⩽0.0001) was observed, reflecting that increases in dosage and time of treatment were associated with decreases in cell growth.

In contrast, treatment with rosiglitazone or ciglitazone did not induce significant cell growth inhibition in any of the tested cell lines (*P*>0.05), even when the highest dosage of treatment (i.e. 50 μM) for the longest duration (i.e. 72 h) was employed (Figure 2C).

### Effect of 15-deoxy-Δ^12,14^-PGJ_2_ on apoptosis and cell proliferation

We tested next whether the cell growth inhibition induced by 15-PGJ_2_ was due to alterations in apoptosis or cell proliferation rates.

Treatment of oral SCCa cells with 10 or 20 μM of 15-PGJ_2_ did not significantly affect the percentage of cells in the S phase of the cell cycle, which remained stable or was only slightly increased; however, an increase in the percentage of cells in the G2 phase of the cell cycle was observed, accompanied by a corresponding reduction of cells in the G1 phase (Table 2

**Table 2:**
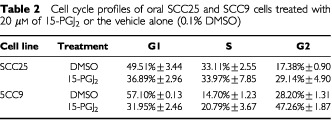
Cell cycle profiles of oral SCC25 and SCC9 cells treated with 20 μM of 15-PGJ_2_ or the vehicle alone (0.1% DMSO)

). These results indicate a relative accumulation of cells in the G2 phase, which may interfere with cell cycle progression. On the other hand, significant 5–8-fold increases in the levels of apoptosis resulted following treatment with 15-deoxy-Δ^12,14^-PGJ_2_ for 72 h at concentrations ranging from 10 to 20 μM (F_2,4_=9.5, *P*⩽0.05) (Figure 3

**Figure 3:**
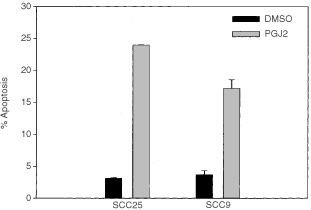
Effect of 15-deoxy-Δ^12,14^-PGJ_2_ on levels of apoptosis of oral SCC25 and SCC9 cells treated with 0.1% DMSO or 20 μM of the drug for 72 h. Treatment with 15-deoxy-Δ^12,14^-PGJ_2_ resulted in a statistically significant increase in apoptosis compared to DMSO-treated control cells. Similar results were obtained from the other oral SCCa cell lines tested.

).

### Effects of PPAR agonists on Stat3 phosphorylation and expression

Because of the critical role of Stat3 in head and neck carcinogenesis (Grandis *et al*, 1998, 2000) and our recent demonstration of sulindac-mediated downmodulation of Stat3 in oral SCCa (Nikitakis *et al*, 2002b), we explored the hypothesis that ligand-mediated PPARγ activation causes changes in Stat3 expression and activation.

**Figure 4:**
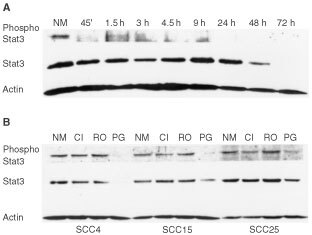
(**A**) 15-deoxy-PGJ_2_ inhibits phosphorylation and production of Stat3 in oral SCCa cells. Oral SCC9 cells were treated with normal medium (NM) or 20 μM 15-deoxy-PGJ_2_ for 45 min, or 1.5, 3, 4.5, 9, 24, 48, 72 h, as indicated. Cells were lysed, blotted with antibody to phosphorylated Stat3 (phospho Stat3), and then sequentially stripped and reprobed with antibodies to Stat3 and to actin. (**B**) 15-deoxy-PGJ_2_, but not rosiglitazone or ciglitazone, inhibits phosphorylation and production of Stat3 in oral SCCa cells. Oral SCCa cells, derived from cell lines SCC4, 15, or 25, were treated for 48 h with normal medium (NM), 50 μM ciglitazone (CI), 50 μM rosiglitazone (RO), or 20 μM 15-deoxy-PGJ_2_ (PG), as indicated. Cells were lysed, blotted with antibody to phosphorylated Stat3 (phospho Stat3), and then sequentially stripped and reprobed with antibodies to Stat3 and to actin.

Forty-five minutes of treatment with 20 μM of 15-PGJ_2_ resulted in a significant reduction of phosphorylated Stat3 in SCC9 cells. Longer treatment, up to 9 h, did not induce further reduction in phosphorylated Stat3 levels. In contrast, phosphorylated Stat3 levels were eliminated after 24, 48 or 72 h of treatment with 20 μM of 15-deoxy-PGJ_2_ (Figure 4A

). Stat3 protein expression levels in SCC9 cells also exhibited a small decrease after 45 min of treatment with 20 μM 15-PGJ_2_. Nonetheless, the decrease in protein expression of Stat3 could not account for the reduction in phosphorylated levels of this protein. Stat3 protein levels were not further reduced as a result of longer, up to 24 h, treatment. However, 48 h of treatment induced further significant reduction of Stat3 expression, which was eliminated after 72 h of treatment (Figure 4A). In accordance with the aforementioned results, phosphorylated Stat3 levels were eliminated and Stat3 protein expression levels were either eliminated or severely reduced by 72 h of 15-deoxy-PGJ_2_ treatment with 20 μM in all tested cell lines (Figure 4B). These effects could not be attributed to a nonspecific reduction of protein expression, as the protein expression levels of actin were not significantly affected by treatment (Figure 4A,B). On the contrary, treatments with rosiglitazone or ciglitazone at 50 μM concentrations failed to affect the phosphorylation or expression of Stat3 (Figure 4B).

Notably, no changes in PPARγ protein expression were induced by either 15-deoxy-PGJ_2_ or thiazolidinediones (Data not shown).

## DISCUSSION

The capacity of PPARγ to promote differentiation, cell cycle withdrawal and apoptosis has encouraged extensive investigation of its potential anticancer activity in multiple types of human cancer (see Introduction). We recently showed that NSAID sulindac upregulates PPARγ expression and activity and relies on PPARγ availability for its antineoplastic activities on oral SCCa (Nikitakis *et al*, 2002a). These observations prompted us to investigate the possibility that oral SCCa cells are also responsive to treatment with direct PPARγ ligands.

15-PGJ_2_, a natural PPARγ ligand, exerted a statistically significant growth inhibitory effect on oral SCCa cells. Significant increases in the levels of apoptosis were observed, suggesting that 15-PGJ_2 _cell growth inhibitory effect is primarily mediated through induction of apoptosis. Nonetheless, accumulation of cells in the G2 phase of the cell cycle was also noted, supporting the concept that interference of 15-PGJ_2_ with cell cycle progression may also contribute to its activity. In contrast, the well-characterised PPARγ ligands rosiglitazone and ciglitazone did not inhibit the cell growth of oral SCCa cells, implying that PPARγ activation is not sufficient for inducing antineoplastic effects on oral SCCa cells. These results raise the possibility that the antineoplastic properties of 15-PGJ_2_ are, at least partially, mediated through PPARγ-independent mechanisms.

PPARγ-independent effects of 15-PGJ_2_ have been previously reported in other systems, including chondrocytes, myofibroblasts, mesangial cells, inflammatory cells and cells of the nervous system (Petrova *et al*, 1999; Castrillo *et al*, 2000; Rossi *et al*, 2000; Straus *et al*, 2000; Boyault *et al*, 2001; Li *et al*, 2001; Janabi, 2002; Ward *et al*, 2002). Several mechanisms have been implicated as responsible for the effects of 15-PGJ_2_ on these cells and may also partly explain its antineoplastic properties. Negative regulation of the NF-κB pathway through inhibition of IκB kinase (IKK) and abrogation of the DNA binding ability of NF-κB, has emerged as a major pathway of PPARγ-independent 15-PGJ_2_ activity (Petrova *et al*, 1999; Castrillo *et al*, 2000; Rossi *et al*, 2000; Straus *et al*, 2000; Boyault *et al*, 2001; Janabi, 2002;). 15-PGJ_2_-mediated NF-κB inhibition has been linked to downregulation of inducible nitric oxide synthase (iNOS) and abrogation of cyclooxygenase-2 (COX-2) transactivation (Petrova *et al*, 1999; Castrillo *et al*, 2000; Rossi *et al*, 2000; Straus *et al*, 2000; Boyault *et al*, 2001; Janabi, 2002). Similarly, Ward *et al* (2002) recently showed that 15-PGJ_2_ exploits PPARγ-independent inhibition of NF-κB activation to induce caspase-dependent apoptosis in granulocytes. Other mediators that have been implicated in PPARγ-independent properties of 15-PGJ_2_ and can potentially have a similar function in cancer cells include AP-1 (Boyault *et al*, 2001), MAP kinase (Harris *et al*, 2002; Lennon *et al*, 2002), and reactive oxygen species (Li *et al*, 2001; Lennon *et al*, 2002). The latter has been shown to act as intermediates for the induction of apoptosis caused by 15-PGJ_2_ in human myofibroblasts (Li *et al*, 2001). Identification of the molecular pathways that mediate the PPARγ-independent antineoplastic effects of 15-PGJ_2_ should be thoroughly addressed in future studies. In this respect, the recent observations of Clay *et al* (2001) that early *de novo* gene expression is necessary for 15-PGJ_2_-induced apoptosis in breast cancer cells may be of particular relevance.

Although our data strongly support the existence of PPARγ-independent effects of 15-PGJ_2_ on oral SCCa cells, recruitment of PPARγ-mediated pathways cannot be ruled out. However, the precise molecular mechanisms that are responsible for the antineoplastic properties of PPARγ are not well understood. An association has been suggested between PPARγ and COX-2, which has also been implicated in various human cancers, including head and neck SCCa (Chan *et al*, 1999; Dannenberg *et al*, 2001). PPARγ activators may inhibit COX-2 expression, possibly through negative interference with NF-κB and/or AP-1 activation (Inoue *et al*, 2000; Subbaramaiah *et al*, 2001; Yang and Frucht, 2001). There is also evidence that supports the function of PPARγ ligands as potent inhibitors of angiogenesis *in vivo* and *in vitro*, providing an additional mechanism that may partially account for the anticancer properties of PPARγ (Bishop-Bailey and Hla, 1999; Xin *et al*, 1999). Finally, cross-talk between PPARγ and other signalling molecules, such as NF-κB, AP-1 and STAT (Ricote *et al*, 1998; Zhou and Waxman, 1999a,b), may contribute significantly to the effects of PPARγ on tumour growth. The possible contribution, if any, of PPARγ activation to growth inhibition induced by 15-PGJ_2_ treatment warrants further exploration.

Constitutive activation of Stat3 plays an important role in the tumorigenesis of various types of human cancer (Catlett-Falcone *et al*, 1999; Fernandes *et al*, 1999), and abrogation of Stat3 signalling has been correlated with stimulation of cell proliferation, prevention of apoptosis and tumour formation (Bromberg *et al*, 1999; Bowman *et al*, 2000). Based on accumulating evidence that Stat3 is upregulated in head and neck SCCa (Grandis *et al*, 1998, 2000), we investigated whether treatment with PPARγ ligands exerts an effect on Stat3 protein expression and Stat3 tyrosine phosphorylation. Treatment of oral SCCa cells with 15-PGJ_2_ at concentrations that cause significant reduction of cell growth resulted in downregulation of both Stat3 expression and phosphorylation. Given that targeting of Stat3 in head and neck SCCa has been linked to significant growth inhibition and induction of apoptosis both *in vitro* and *in vivo* (Grandis *et al*, 1998, 2000), our results suggest that the ability of 15-PGJ_2_ to downregulate Stat3 may be aetiologically related to its growth inhibitory and apoptotic effects. Interestingly, the mode of Stat3 downregulation (i.e. reduction of Stat3 phosphorylated levels after 45 min and elimination of both Stat3 phosphorylated and unphosphorylated levels after 72 h) was very similar to that induced by sulindac sulphide in the same cell lines (Nikitakis *et al*, 2002b). Although sulindac's growth inhibitory effect was dependent on PPARγ availability, its ability to induce Stat3 downmodulation was independent of its ability to act as a PPARγ ligand. Similarly, the inability of PPARγ activation trough rosiglitizone and ciglitazone stimulation to affect the phosphorylation and expression levels of Stat3 entails that activation of PPARγ-independent mechanisms is necessary for 15-PGJ_2_-mediated Stat3 downmodulation.

Both PPARγ-dependent and PPARγ-independent mechanisms may come into play during Stat3 downregulation by 15-PGJ_2_. Direct protein–protein interactions or indirect mechanisms, such as competition for common co-activators or modulation of inhibitors of transcriptional activity, have been suggested as possible mediators of STAT-PPAR cross-talk and could account for the Stat3 inhibitory effect of PPARγ activation (Zhou and Waxman, 1999a,b). Cytokine stimulation results in phosphorylation of STATs through the mediation of the Janus kinase (JAK) family of protein tyrosine kinases (Darnell *et al*, 1994). Accordingly, the recently proposed association between PPARγ and cytokine expression may represent another possible connection between Stat3 and PPARγ. Indeed, PPARγ agonists have been shown to suppress monocyte elaboration of inflammatory cytokines (Jiang *et al*, 1998) and to inhibit IL-1β-induced expression of IL-8 in colon cancer cell lines (Su *et al*, 1999). 15-PGJ_2_-mediated inhibition of cytokine production and function may also ensue through PPARγ-independent pathways; for example, 15-PGJ_2_ has been shown to modulate IL-1β effects in human chondrocytes (Boyault *et al*, 2001) and to inhibit TNF-α and IL-6 production in human macrophages without PPARγ mediation. Inhibition of cytokine expression by 15-PGJ_2_ would conceivably result in decreased activation of STAT molecules that mediate the transduction of the cytokine signal from the cell surface to the nucleus. Head and neck cancer cells have been shown to express a variety of pro-inflammatory and pro-angiogenic cytokines (Chen *et al*, 1999; Ondrey *et al*, 1999) and to respond to IL-6 stimulation with upregulation of Stat3 phosphorylation and promotion of cell growth; reversal of this process could participate in the observed effects of 15-PGJ_2_ on oral SCCa cells. In that aberrant tumour growth factor-α/epidermal growth factor receptor signalling has been demonstrated to play a major role in Stat3 constitutive activation of head and neck SCCa cells, possible interference of 15-PGJ_2_ with this pathway, at the level of the ligand or the receptor, constitutes another distinct possibility.

In summary, we demonstrated that 15-PGJ_2_, a specific natural PPARγ ligand, inhibits growth of oral SCCa cells, which may be related to its capacity to downregulate the oncogene Stat3. The ineffectiveness of rosiglitazone and ciglitazone to cause similar effects strongly suggests that 15-PGJ_2_ effects are at least partly mediated through PPARγ-independent mechanisms. Delineation of these mechanisms, as well as determination of the potential contribution of PPARγ activation, not only will provide an explanation for 15-PGJ_2_ anticancer qualities but it will also enhance our understanding of critical signalling pathways for oral SCCa carcinogenesis.
